# Amplicon DNA Melting Analysis for the Simultaneous Detection of *Brucella spp* and *Mycobacterium tuberculosis* Complex. Potential Use in Rapid Differential Diagnosis between Extrapulmonary Tuberculosis and Focal Complications of Brucellosis

**DOI:** 10.1371/journal.pone.0058353

**Published:** 2013-03-08

**Authors:** Rocio Sanjuan-Jimenez, Juan D. Colmenero, Pilar Bermúdez, Antonio Alonso, Pilar Morata

**Affiliations:** 1 Biochemistry, Molecular Biology and Immunology Department, Faculty of Medicine, University of Malaga, Malaga, Spain; 2 Infectious Diseases Service, Carlos Haya University Hospital, Malaga, Spain; 3 Microbiology Service, Carlos Haya University Hospital, Malaga, Spain; Cornell University, United States of America

## Abstract

Some sites of extrapulmonary tuberculosis and focal complications of brucellosis are very difficult to differentiate clinically, radiologically, and even histopathologically. Conventional microbiological methods for the diagnosis of extrapulmonary tuberculosis and complicated brucellosis not only lack adequate sensitivity, they are also time consuming, which could lead to an unfavourable prognosis. The aim of this work was to develop a multiplex real-time PCR assay based on SYBR Green I to simultaneously detect *Brucella* spp and *Mycobacterium tuberculosis* complex and evaluate the efficacy of the technique with different candidate genes. The IS711, bcsp31 and omp2a genes were used for the identification of *Brucella* spp and the IS6110, senX3-regX3 and cfp31 genes were targeted for the detection of the *M. tuberculosis* complex. As a result of the different combinations of primers, nine different reactions were evaluated. A test was defined as positive only when the gene combinations were capable of co-amplifying both pathogens in a single reaction tube and showed distinguishable melting temperatures for each microorganism. According to the melting analysis, only three combinations of amplicons (senX3-regX3+bcsp31, senX3-regX3+IS711 and IS6110+IS711) were visible. Detection limits of senX3-regX3+bcsp31 and senX3-regX3+IS711 were of 2 and 3 genome equivalents for *M. tuberculosis* complex and *Brucella* while for IS6110+IS711 they were of 200 and 300 genome equivalents, respectively. The three assays correctly identified all the samples, showing negative results for the control patients. The presence of multicopy elements and GC content were the components most influencing the efficiency of the test; this should be taken into account when designing a multiplex-based SYBR Green I assay. In conclusion, multiplex real time PCR assays based on the targets senX3-regX3+bcsp31 and senX3-regX3+IS711 using SYBR Green I are highly sensitive and reproducible. This may therefore be a practical approach for the rapid differential diagnosis between extrapulmonary tuberculosis and complicated brucellosis.

## Introduction

Tuberculosis is a major worldwide cause of death and disease. An estimated 2 billion persons, one-third of the world’s population, are infected with *Mycobacterium tuberculosis* complex (MTC). This enormous reservoir results in over 8 million new cases and 2 million deaths from tuberculosis each year. On the other hand, brucellosis is an emerging and re-emerging disease which remains the most common bacterial zoonosis in the world, with over half a million new cases annually [1]-[2]. Both tuberculosis and brucellosis are systemic granulomatous diseases that can affect any organ or system. Some extrapulmonary tuberculosis and focal complications of Brucellosis, such as meningitis, vertebral osteomyelitis, arthritis or orchiepididimitis, are difficult to differentiate clinically and radiologically and their diagnosis requires microbiological confirmation [3]. MTC and Brucella spp are slow-growing microorganisms, and their isolation requires incubation for several days or weeks. These two situations mean that diagnosis is often delayed, which may have a negative effect on prognosis. In both diseases, rapid diagnosis is necessary for the timely initiation of the correct antimicrobial therapy.

Molecular methods, especially PCR, are more rapid and sensitive than cultures in diagnosing tuberculosis and brucellosis. The multiplex real time polymerase chain reaction (M RT-PCR) technique has proven to be a very useful strategy for the rapid differential diagnosis of syndromes produced by different microorganisms. Our group has recently developed a M RT-PCR able to amplify *M. tuberculosis* complex and *Brucella* spp simultaneously using FRET probes [4].

SYBR Green I (SG) is an intercalating agent that binds non-specifically to double-stranded DNA. Its low cost compared with that of specific fluorescent probes makes it a very attractive candidate for clinical laboratories with limited resources. The aim, therefore, of this experimental study was to develop and optimize a multiplex real-time PCR assay using different specific targets able to detect and differentiate MTC and *Brucella* spp by analyzing the melting curves obtained when using an intercalating agent, such as SG.

## Materials and Methods

### Bacterial Strains, Culture Media and Growth Conditions

The strains of *Brucella* spp used in this study (*B. abortus* B-19, *B. melitensis* Rev-1, *B. melitensis* 1 16 M, *B. abortus* 6 870) were supplied by the Microbiology Department of the Faculty of Medicine at Valladolid University, except for the vaccine strains B-19 and Rev-1, kindly provided by the Agriculture Department of the Andalusia Regional Government. These strains were cultured on Brucella agar (Difco, USA) and incubated at 37°C with 5% CO_2_ for 48 h. The strains of MTC were provided by the American Types Culture Collection (*M. tuberculosis* H37Rv ATCC 27294 and *M. bovis* ATCC 19210). Mycobacterial strains were cultured on Lowenstein-Jensen medium (Biomedics, Spain) and incubated at 37°C for 2–4 weeks in order to obtain sufficient bacterial growth for later extraction of genomic DNA. All procedures were performed in a biosafety cabinet class II B3.

### DNA Extraction

#### DNA extraction from bacteria

The strains of Brucella were inactivated with equal parts of pure acetone and 0.85% NaCl held in suspension at 4°C overnight and the strains of MTC were inactivated by heat at 85°C for 15 minutes. Inactivated microorganisms were washed and resuspended in TE 1X (10 mM Tris/HCL at pH 8, 1 mM EDTA). Genomic DNA was isolated by the CTAB method as described previously [5].

#### Extraction of DNA from clinical samples

DNA from different clinical samples was extracted from an approximate volume of 350 µl using BioRobot EZ1 Advanced system with EZ1 DSP DNA Tissue Kit (Quiagen, UK), based on the magnetic-particle technology, and eluted in 50 µl of TE 1X. The concentration and purity of DNA were estimated by measuring the absorbance at 260 nm and 280 nm with a Nanodrop ND-1000 spectrophotometer (ThermoFisher, USA). All DNA samples were stored at −20°C until required for analysis.

### Real-time PCR Primer Design

In order to identify possible targets for detection of the MTC and *Brucella* spp, extensive literature and nucleotide sequence searches were performed in the databases. For detection of *Brucella* spp a number of housekeeping genes that are highly conserved in the genus were evaluated, and for detection of MTC, targets present in all members of MTC but absent in the other species of *Mycobacterium* genus were evaluated. For each microorganism three candidate genes were chosen: bcsp31, IS711, and omp2a for *Brucella spp;* and cfp32, IS6110, and senX3-regX3 for MTC. The sequences obtained were aligned using the Blastn program (Basic Local Alignment Search Tool Nucleotide) and primers were designed using Beacon Designer software (PREMIER Biosoft, USA) (Table 1). The oligonucleotides were designed in accordance with the general recommendations. All primers were supplied by Sigma Aldrich (USA).

**Table 1:** Primer sequences used for amplification by real-time PCR.

Target	Primer	Sequence (5′–3′)	Position	Size (bp)	Genbank Access No
bcsp31	bcsp31f	GCATTCTTCACATCCAGG	348–366		
	bcsp31r	CACCGCATTCCATTATTCT	517–498	151	M20404
cfp32	Rv0577f	GCCCAAGAGAAGCGAATA	3–21		
	Rv0577r	GAACAACGATGTGTAGAACT	99–79	97	NC000962
omp2a	Omp2af	ACGCCGAACCAGAACTAC	831–849		
	Omp2ar	TTCCACTCGCCACCAAAT	1042–1024	212	U26438
IS711	IS711f	TACAAGGAACGCCATCAGA	691–710		
	IS711r	GCATTCAACGCAACCAGA	832–814	142	AE017223
IS6110	IS6110f	TCAAGGAGCACATCAGCC	553–571		
	IS6110r	TCACGGTTCAGGGTTAGC	634–616	82	BX842574
*sen*X3-*reg*X3	M1f	CGGCTAATCACGACGGCAC	1114–1132		
	M3r	CTCTTCCTCTCGTTGTGACCTGTT	1277–1254	164	BX842573

### Real-time Assay

To optimize the reaction parameters, all tests were performed initially in an individual format. These real time PCR assays and the parameters used in the design of the primers were taken into account to facilitate subsequent multiplex reactions, once the optimization and sensitivity were completed. A total of nine M RT-PCR reactions were developed and evaluated, as a result of the different gene combinations. Amplifications and melting curve analyses were performed on the LightCycler 2.0 Instrument (Roche Diagnostic, Indianapolis, IN) using the LC® FastStart DNA Master SYBR Green I kit (Roche Molecular Biochemicals, Mannheim, Germany). The multiplex amplification mixture included 1X master mix, 3–4 mM MgCl_2_, 0.5–0.6 µM primers, 4 µl of template DNA (2 µl of each microorganism) at different concentrations from 10^7^ to 10^1^ fg and nuclease free dH_2_O adjusted to a final volume of 20 µl. Each run included positive controls consisting of dilutions of *Brucella* spp and MTC DNA, and negative controls with all the elements of the reaction mixture except template DNA.

The cycling conditions consisted of an initial hold at 95°C for 10 min and 45 cycles of 95°C for 10 s, 60°C for 5 s, and 72°C for 8 s with programmed transitions of 20°C/s. Following amplification, melting curves were acquired on the SYBR channel by heating momentarily at 95°C, cooling to 65°C and collecting fluorescence continuously at a ramping rate of 0.1°C/s until 95°C. To minimize experimental variability the Ct values, the threshold cycle where the fluorescence signal rises significantly above background in the exponential phase of the amplification, were determined by the second derivative maximum method.

### Analytical Sensitivity

The sensitivity was evaluated by decreasing DNA quantities from *B. abortus* B-19 and *M. bovis* ATCC 19210, either independently or jointly, by serial decimal dilutions from 10^7^ to 10^1^ fg. These strains were chosen due to low copy numbers of their multicopy element. The number of genome equivalents (GE) was determined based on the genome size of *B. abortus* S-19 (3.283.936 bp) and *M. tuberculosis* H37Rv (4.411.532 bp), approximately equivalent to 3.37 fg and 4.53 fg DNA per cell respectively.

### Repeatability and Reproducibility

Intra-assay variability was determined by amplification in the same run of ten samples for each serial dilution. For inter-assay variability each dilution was tested in quadruplicate on five alternate days. Repeatability and reproducibility were estimated by computing the coefficient of variation.

### Assessment of Interference by Human DNA

To assess the possible interference by human DNA (hDNA) in the amplification reaction, DNA obtained from blood donors and extracted as described above were added to the reaction mixture. The data obtained on the multiplex real time PCRs were compared with and without the addition of 100, 150, 250, 500 and 700 ng of hDNA.

### Clinical Samples

Ten non-blood clinical specimens from 5 patients with different focal complications of brucellosis and 5 patients with extrapulmonary tuberculosis were studied by M RT-PCR assay. Control samples were obtained from 5 patients with other disorders initially involved in the differential diagnosis of brucellosis or extrapulmonary tuberculosis. These samples came from vertebral tissue (3 patients), cerebrospinal fluid (CSF) (3 patients), synovial fluid (3 patients), tissue or pus from hepatosplenic abscesses (2 patients), pleural fluid (2 patients), a psoas abscess (1 patient), and pericardial tissue (1 patient).

The diagnosis of brucellosis was established according to one of the following criteria: first, the isolation of Brucella from any clinical specimen and second, the presence of a compatible clinical picture, together with the demonstration of specific antibodies at significant titres or seroconversion. Significant titres were considered to be a Wright’s seroagglutination test (SAT) ≥1∶160 or immunocapture-agglutination test ≥1∶320. The diagnosis of tuberculosis was based on the isolation of any species of the MTC, or the presence of caseating granulomas, with or without acid-fast bacilli, in patients with compatible clinical findings and a good response to antituberculous treatment.

### Ethics Statement

All patients gave written informed consent prior to collection of biological samples. The use of samples for research was approved by the Ethics Committee of Carlos Haya University Hospital, Malaga, Spain.

### Confirmation of M RT-PCR Product

To confirm the identities of the amplified fragments, the M RT-PCR products of *Brucella* spp and MTC were sequenced. The ABI PRISM Big Dye Terminator Cycle sequencing reaction kit v. 3.0 (Applied Biosystems, Madrid, Spain) was used for the sequencing reactions. Sequence analysis was by capillary electrophoresis in an ABI PRISM, model 3100 automated sequencer (Applied Biosystems). When required, samples were analyzed by 2% agarose gel electrophoresis and ethidium bromide staining using standard methods.

### Statistical Analysis

Quantitative variables are represented as mean ± standard deviation and qualitative variables as percentages. The results were compiled in a database created with Microsoft Office software Excel 2007 (Microsoft®, Redmond). The amplification sensitivity of the assay was defined as the percentage of results correctly identified as positive among all the replicas made and the specificity of the results as the percentage of results correctly identified as negative from the total number of samples processed. The standard curves obtained for the individual real-time PCR assays, regression equations and correlation coefficients were generated in Microsoft® Excel 2007. To evaluate significant differences between the Tm and Ct values obtained from pure cultures and experimentally infected biological samples, one-way analysis of variance (ANOVA) was performed using the Statistical Program for the Social Sciences version 13.0 (SPSS ® Inc., Chicago, IL). A *p* value <.05 was considered statistically significant.

## Results

### Monoplex PCR Assays

**Figure 1:**
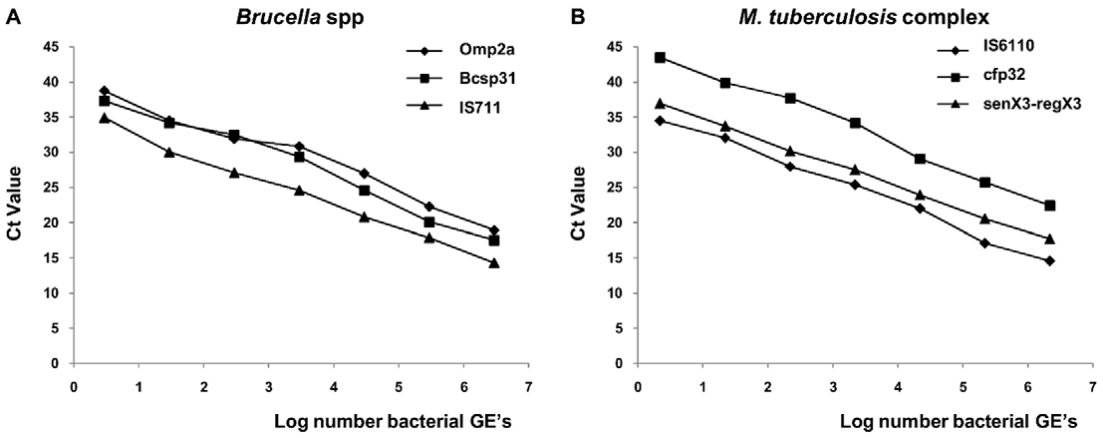
Standard monoplex assay curves. Panel A. Comparison between standard curves of the three real-time PCR for *Brucella* spp. Panel B. Comparison between standard curves of the three real-time PCR for *M. tuberculosis* complex.

**Figure 2:**
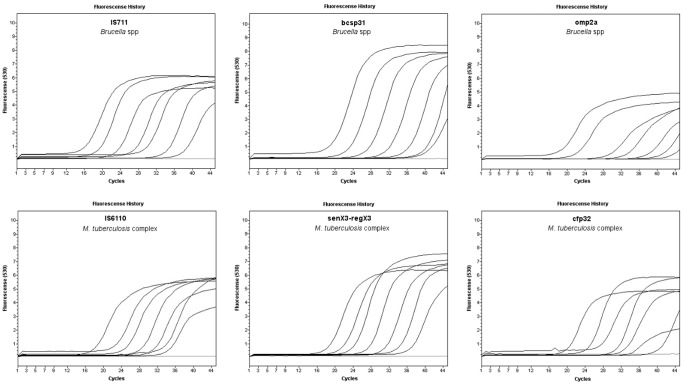
Representation of SG fluorescence history versus cycle number of targets IS711, bcsp31, omp2a, IS6110, senX3-regX3 and cfp32. DNA was quantified and serial ten-fold dilutions were prepared from 10^7 ^fg to 10^1^ fg from *B. abortus* B-19 and *M. bovis* ATCC 19212.

Decimal serial dilutions of DNA from *B. abortus* B-19 and *M. bovis* ATCC 19210 were amplified in triplicate to generate a standard quantification curve for each monoplex assay. For all the assays generated, standard curves and linear regression equations for each target (a strong linear relationship between the log of the genome number and the Ct values) were obtained (Figure 1). For the identification of Brucella spp, the slopes of the standard curves were most efficient for IS711 (E = 2.00±0.03), followed by bcsp31 (E = 1.96±0.07) and omp2a (E = 1.90±0.03). The detection limit was 3×10^0^ GEs in all cases. The linear regression equations were −3.30 log [GE] +35.67 (R^2^ = 0.99), −3.41 log [GE] +39.74 (R^2^ = 0.98) and −3.57 log [GE] +40.17 (R^2^ = 0.98) for IS771, bcsp31 and omp2a, respectively. Of the three targets tested for identification of MTC, the highest slopes of the standard curves were, in order, senX3-regX3 (E = 2.03±0.06), IS6110 (E = 1.96±0.06) and cfp32 (E = 1.87±0.08). The detection limit was 2×10^0^ genomes per reaction. The linear regression equations were −3.23 log [GE] +37.97 (R^2^ = 0.99), −3.42 log [GE] +36.20 (R^2^ = 0.99), and −3.67 log [GE] +45.59 (R^2^ = 0.99). A representation of real time PCR SYBR Green I fluorescence history versus cycle number for each candidate target is shown in Figure 2.

### Multiplex Real Time

**Figure 3:**
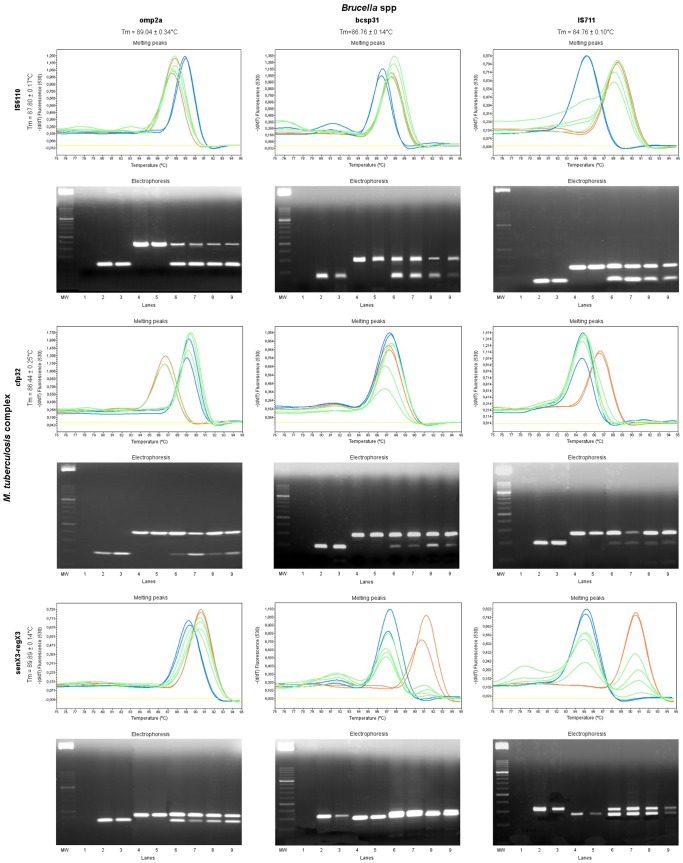
Evaluation of the M RT-PCR assay and analysis of amplicons by agarose gel electrophoresis. A total of 9 M RT-PCR reactions based on SG were evaluated using the different primer combinations. The omp2a, bcsp31 and IS711 genes were used for the identification of *Brucella* spp and the IS6110, cfp32 and senX3-regX3 genes were targeted for the detection of members of the MTC. The orange lines represent the positive controls of *M. bovis*, blue lines indicate the positive control of *Brucella* spp, a yellow line represents the negative control and green lines a mixture of both pathogens at different concentrations. Below each image of melting peaks is shown the corresponding electrophoresis. Lanes: Mw, molecular size DNA ladder XIII; 1, negative controls; 2 to 3, positive controls for *M*. *bovis*; 4 to 5, positive controls for *B. abortus* B-19; 6 to 9, multiplex DNA of MTC and *Brucella*.

Using different gene combinations, a total of 9 M RT-PCR reactions were performed. The initial criteria for selection were the ability to detect both the pathogens simultaneously in a single reaction with clearly differentiated melting temperature values. The electrophoretic analysis of amplicons on agarose gel showed that both targets were correctly amplified for all the gene combinations. However, analysis of the melting curves showed that an appropriate simultaneous amplification was only achieved with three of the combinations tested; senX3-regX3+bcsp31, senX3-regX3+IS711, and IS6110+IS711 (Figure 3).

### Analytical Sensitivity

The detection limit of the senX3-regX3+bcsp31 and senX3-regX3+IS711 combinations was identical to that of their monoplex format assays. By contrast, the combination using the insertion elements IS6110+IS711 decreased two orders of magnitude over the individual results.

### Repeatability and Reproducibility

The analyses of the repeatability and reproducibility were performed with DNA concentrations of 10^7^ and 10^1^ fg for each microorganism. The mean coefficients of variation (CV) for the intra- and inter-assay of multiplex senX3-regX3+bcsp31, senX3-regX3+IS711 and IS6110+IS711 are shown in Table 2. The CVs obtained for senX3-regX3+bcsp31 were lower than for the combination senX3-regX3+IS711. For multiplex IS6110+IS711, practically all the results were obtained only with the IS6110 target.

**Table 2:** Values of reproducibility and repeatability for the combinations senX3-regX3+bcsp31, senX3-regX3+IS711 and IS6110+IS711.

	*sen*X3-*reg*X3+bcsp31 assay					
	Ct (x¯±SD)		CV (%)		Sensibility (%)	
	100 fg	10 fg	100 fg	10 fg	100 fg	10 fg
**Repeatability**	34.40±0.36	35.84±0.42	1.04	1.17	100.00	90.00
**Reproducibility**	34.18±1.93	35.37±2.65	5.64	7.49	88.46	88.88
	***sen*** **X3-** ***reg*** **X3+IS711 assay**					
	**Ct (x¯±SD)**		**CV (%)**		**Sensibility (%)**	
	**100** **fg**	**10** **fg**	**100** **fg**	**10** **fg**	**100** **fg**	**10** **fg**
**Repeatability**	29.86±1.57	32.22±2.36	5.25	7.32	100.00	90.00
**Reproducibility**	28.90±1.93	31.54±2.90	6.68	9.19	96.55	84.84
	**IS6110+IS711 assay**					
	**Ct (x¯±SD)**		**CV (%)**		**Sensibility (%)**	
	**100** **fg**	**10** **fg**	**100** **fg**	**10** **fg**	**100** **fg**	**10** **fg**
**Repeatability**	28.83±0.49	30.58±0.49	1.60	7.32	00.00*	00.00*
**Reproducibility**	28.29±0.91	30.74±1.52	3.21	4.94	34.00	00.00*

* Only amplification of *M. bovis*.

### Assessment of Interference by Human DNA

**Figure 4:**
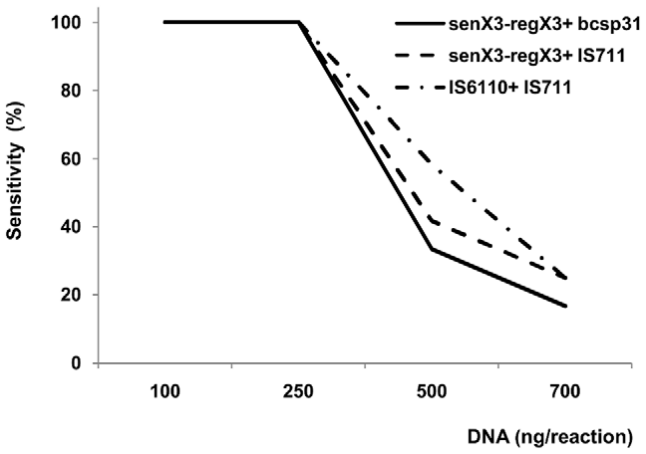
Sensitivities of detection for amplification assay with addition of human DNA.

In all cases, the amplification sensitivity decreased with increasing amounts of total DNA in the reaction. Thus, concentrations above 250 ng of DNA reduced the chance of amplifying all bacterial dilutions tested (Figure 4). The possible interference in the detection range was assessed by amplification with serial dilutions of bacterial DNA and the addition of 150 ng of hDNA. For the combinations senX3-regX3+bcsp31 and senX3-regX3+IS711, the melting temperature values of MTC, melting temperatures of *Brucella* and Ct values were compared with those obtained in pure cultures by one-way ANOVA. The Tm and Ct values did not differ significantly between the different conditions (Table 3).

**Table 3:** Values of Tm and Ct obtained in pure cultures and experimentally infected biological samples with hDNA for multiplex assays.

	senX3-regX3+bcsp31					
fg	Pure cultures DNA			Experimental infection DNA		
	Tm		Ct	Tm		Ct
	*B.abortus*	*M.bovis*		*B.abortus*	*M.bovis*	
10^7^	86.35±0.72	90.34±0.42	19.59±0.63	86.40±0.57	90.15±0.07	17.85±2.33
10^6^	85.75±0.21	90.22±0.50	20.84±0.13	86.40±0.11	90.08±0.02	18.55±0.66
10^5^	85.64±0.43	90.21±0.32	23.58±0.12	85.89±0.04	90.14±0.13	22.26±0.54
10^4^	85.97±0.29	90.17±0.16	26.77±0.16	86.03±0.23	89.89±0.02	26.13±0.75
10^3^	85.73±0.33	90.24±0.05	30.90±0.37	86.29±0.36	89.97±0.02	28.63±0.66
10^2^	85.83±0.30	90.11±0.15	34.58±0.92	85.96±0.52	89.89±0.26	31.21±0.38
10^1^	86.29±0.49	89.87±0.37	36.72±1.29	86.16±0.19	90.14±0.02	33.30±0.54
	**senX3-regX3+IS711**					
**fg**	**Pure cultures DNA**			**Experimental infection DNA**		
	**Tm**		**Ct**	**Tm**		**Ct**
	***B.abortus***	***M.bovis***		***B.abortus***	***M.bovis***	
10^7^	84.60±0.33	90.03±0.29	20.01±0.57	84.34±0.39	90.14±0.08	15.40±1.32
10^6^	84.47±0.36	90.14±0.36	22.07±0.66	84.20±0.19	90.02±0.25	19.90±0.93
10^5^	84.20±0.25	90.27±0.30	23.97±0.68	84.43±0.54	90.23±0.11	21.18±0.33
10^4^	84.49±0.40	90.45±0.41	26.72±0.31	84.73±0.19	90.33±0.09	25.02±0.30
10^3^	84.15±0.12	90.41±0.07	28.40±0.19	84.63±0.12	90.19±0.12	25.67±0.14
10^2^	84.51±0.33	89.97±0.61	30.15±0.31	84.64±0.21	90.19±0.03	27.86±0.38
10^1^	84.66±0.33	89.56±1.33	32.42±0.27	84.60±0.21	90.12±0.07	29.36±0.28
	**IS6110+IS711**					
**fg**	**Pure cultures DNA**			**Experimental infection DNA**		
	**Tm**		**Ct**	**Tm**		**Ct**
	***B.abortus***	***M.bovis***		***B.abortus***	***M.bovis***	
10^7^	–	87.57±0,19	14.82±1.70	–	87.53±0.77	19.20±7.96
10^6^	84.30±0.28	87.70±0,10	18,46±1,10	–	87.22±0.64	19.87±1.66
10^5^	83.95±0.62	87.77±0,66	20.22±1,29	–	87.52±0.46	24.11±2.13
10^4^	84.16±0.09	87.60±0,07	24.30±0,40	–	87.31±0.47	26.42±2.91
10^3^	83.50±0.49	87.59±0,10	25.17±2.04	83.81±0.51	86.96±0.10	27.12±1.94
10^2^	–	87.46±0.40	28,56±0.10	83.61±0.73	86.59±0.32	28.89±0.71
10^1^	–	87.43±0.14	30,58±0.03	–	86.74±0.39	29.97±4.65

### Preliminary Clinical Results

**Figure 5:**
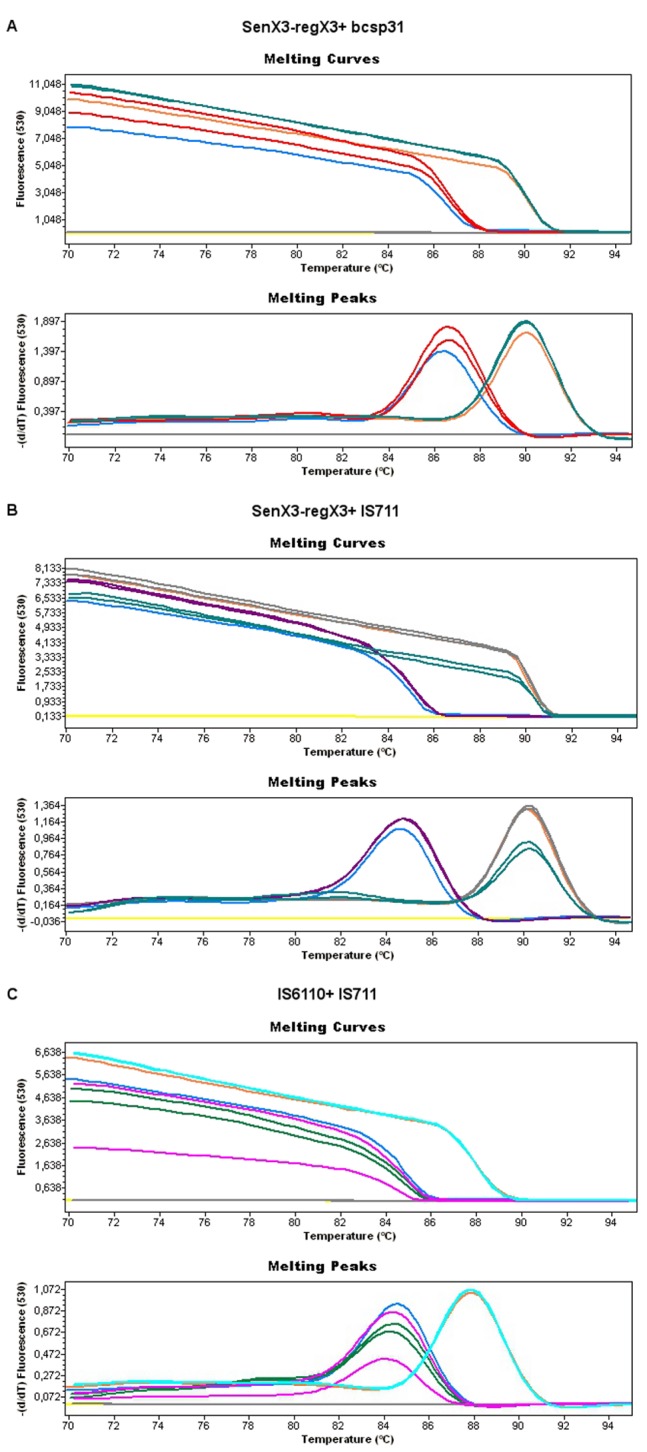
Results of M RT-PCR assays with clinical samples for gene combinations senX3-regX3+bcsp31 (A), senX3-regX3+IS711 (B) and IS6110+IS711 (C). Blue lines, positive control of *B. abortus*; orange lines, positive controls of *M. tuberculosis*; yellow lines, negative controls. Panel A. Red lines, synovial fluid from a patient with brucellosis arthritis; green lines, vertebral tissue from a patient with tuberculous vertebral osteomyelitis; and grey lines, CFS from a patient with neurosyphilis. Panel B. Purple lines, samples from a patient with Brucellar hepatosplenic abscesses; and grey and green lines, pericardial tissue and CFS from a patient with tuberculosis meningitis. Panel C. Pink and green lines, synovial fluid and CFS from two patients with brucellosis; turquoise lines, vertebral tissue from a patient with vertebral tuberculosis; and grey lines, vertebral tissue from a patient with vertebral osteomyelitis caused by *Mycobacterium xenopi*.

M RT-PCR assays identified Brucella DNA in the five samples and with the three gene combinations. Equally, M RT-PCR assays also detected DNA of MTC in the five samples from tuberculosis. All the multiplex combinations were negative in all the control samples (Figure 5). The average Tm values for patients with brucellosis were 86.29±0.49°C for multiplex senX3-regX3+bcsp31, 84.85±0.71°C for senX3-regX3+IS711, and 84.53±0.46°C for IS6110+IS711. For samples from patients with extrapulmonary tuberculosis these temperatures were 90.36±0.52°C, 90.54±0.31°C and 87.68±0.21°C, respectively. In all cases the melting temperature analysis confirmed the nature of the amplified products. Moreover, sequencing of amplified products of some selected patients perfectly matched the DNA sequences of targets corresponding to *M. bovis* and *B. abortus*.

## Discussion

The application of molecular techniques for the rapid diagnosis and monitoring of tuberculosis and brucellosis has generated great interest in recent years, due to the limitations of conventional diagnostic tests. Most clinical laboratories still base the microbiological diagnosis of tuberculosis on microscopy and culture and in the case of brucellosis on serological tests and cultures of blood or other clinical samples. For both microorganisms cultures are time consuming and require direct sample handling, which poses a risk of infection to laboratory personnel and a delay in appropriate antibacterial therapy. The M RT-PCR is a variant of the real-time PCR that has been applied successfully in many areas of DNA analysis, including infectious diseases [6]–[8]. This strategy is especially useful in situations where various microorganisms cause similar clinical syndromes.

SG is a double-strand-specific intercalating DNA dye that allows monitoring of product formation and melting curve analysis, generating a characteristic melting temperature for each amplicon which is equivalent to the detection of a specific size of DNA by electrophoresis. As with other nonspecific intercalating agents, when SG is used in a M RT-PCR reaction, amplicon discrimination is possible if the Tm values differ sufficiently. Thus, the Tm can be used for clinical diagnosis under specific conditions of assay reproducibility, since the values vary depending on factors such as the concentration of MgCl_2_, SG, DNA, and the rate of transition melting [9]–[10].

In the present study, we designed and compared three molecular targets (IS711, bcsp31 and omp2a) of *Brucella* and three targets of MTC (IS6110, cfp31 and senX3-regX3) for their qualitative detection by monoplex real-time PCR. We attempted to optimize the amplification conditions and MgCl_2_ and primer concentrations in order to achieve two objectives; first, to obtain the highest possible efficiency with the designed primers and second, to facilitate the subsequent multiplexing. Under the conditions employed, we found no differences in the detection limit of the three assays for either *Brucella* or for MTC. However, the presence of different copy numbers in microorganism genomes for the insertion elements suggests that the detection limits for these targets depends on each species and biotype. The data recorded in this study correspond to species with lower copy numbers present in the genome.

Various studies have evaluated the detection limit of real-time PCR assays for the detection of Brucella using hybridization probes [11]–[13], and only two SG based studies have been found for the target bcsp31 [14] and the species-specific IS711-alkB [15]. Moreover, few studies have evaluated the detection limit of real-time PCR assays in different formats, and only for the detection of specific species of the MTC [16]–[17]. The detection limits of the assays performed in this study were similar to or greater than the studies described.

Of the nine multiplex assays based on different gene combinations, Tm analysis showed that in six combinations only one of the targets used was visible in the multiplex real time PCR reaction; for the remaining three combinations, senX3-regX3+bcsp31, senX3-regX3+IS711 and IS6110+IS711, both amplicons were always detected, with melting temperatures that were sufficiently differentiated, approximately 3.49°C, 5.70°C, and 3.57°C, respectively. However, the analysis of the products on agarose gels revealed that in all nine gene combinations both gene sequences were being amplified.

The percentage GC content of the amplicons IS711, bcsp31, omp2a, IS6110, cfp32, and senX3-regX3 was 47.18%, 50.30%, 57.07%, 60.97%, 55.67%, and 62.80%, respectively. Of the six gene combinations that did not present two clearly defined peaks, in the multiplex cases with single copy elements (cfp32+omp2a, cfp32+bcsp31 and senX3-regX3+omp2a) the amplicons not detected by melting analysis corresponded to the lowest Tm and GC percentage, while in the reactions with a multicopy element (IS6110+omp2a, IS6110+bcsp31 and cfp32+IS711) the amplicon detected coincided with the multicopy elements. Except for the combination cfp32+IS711, the multicopy element coincided with the highest Tm and GC content.

Finally, in those conditions where the preselected gene combinations (senX3-regX3+bcsp31, senX3-regX3+IS711 and IS6110+IS711) failed to detect both microorganisms simultaneously (e.g., with high concentrations of hDNA), the amplicon not visible after melting analysis also had the lowest Tm and GC content, even when the amplicon did not match the multicopy element.

Previous genotyping studies by Gundry et al. and Witter et al. [10], [18] showed that SG did not differ between homoduplex and heteroduplex products and a Tm only appeared for all the genotypes after melting analysis. In comparison with other fluorescent indicators, SG favoured the products that melted at higher temperatures and could be released and redistributed from the amplicons with a lower Tm to higher Tm during melting. The assay described by Giglio et al. [19] also showed similar discrepancies in the DNA melting curve analysis in multiplex reactions with SG. The authors showed that SG appears to have a preferential binding for GC-rich areas and of a larger size, which together with the redistribution of the dye could explain the lack of detection of the amplicon with the lower Tm.

In our study, the products with a higher GC content were not always the larger. The high GC content in the *Mycobacterium* genome may explain why the smaller fragments of DNA have greater GC percentages than the larger fragments.

The detection limits of the three combinations finally selected; senX3-regX3+bcsp31, senX3-regX3+IS711 and IS6110+IS711, were 10^1^, 10^1^ and 10^3^ fg, respectively. The first two had identical sensitivity in the individual and multiplex formats. However, with the insertion element combination the detection limit dropped two orders of magnitude between the individual and multiplex formats. Furthermore, the simultaneous detection of IS6110+IS711 was achieved in only 75% of the reactions, compared with 100% for the other two gene combinations. In the remaining 25% of the reactions an amplicon was only observed for MTC. Equally, with dilutions below 10^3^ fg and above 10^6^ fg of DNA amplifications were only detected of IS6110. This may be due to the number of copies per genome of the target in the multiplex reaction, which could cause the saturation phase of the PCR to be achieved faster. In addition, the fluorescence emitted by the reaction was lower than for the other two amplification reactions, followed by senX3-regX3+IS711 (1 and 7 copies) and senX3-regX3+bcsp31 (1 copy in both). Increasing the concentration of SG in the reaction mixture improved the detection sensitivity of the combination IS6110+ IS711, like the other less efficient combinations (data not shown).

Given that the bacterial inoculums found in clinical samples from patients with extrapulmonary tuberculosis and focal complications of brucellosis are usually very low, the detection capability of multiplex real-time PCR able to be used in clinical diagnosis should be very high. The sensitivity of senX3-regX3+bcsp31 and senX3-regX3+IS711, of approximately 3 and 2 genome equivalents of Brucella and MTC, covers an inoculum so small that it should be present in any clinical specimen from a patient with extrapulmonary tuberculosis or focal involvement of brucellosis [4].

Under the conditions used, the accuracy of M RT-PCR assays can be considered high, since intra-assay and inter-assay variations of Ct were always lower than 10%.

For the detection of 10^2^ and 10^1^ fg of DNA in the senX3-regX3+bcsp31 assay, the intra-assay variability was about 1% and inter-assay variability less than 8%. In the senX3-regX3+IS711 assay, the intra-assay variability was less than 8% and the inter-assay variability below 10%. The difference in the variability between the two combinations may be related to the number of copies of the molecular targets in the genome. The presence of a total of 8 copies per genome in the combination senX3-regX3+IS711, compared with 2 copies in senX3-regX3+bcsp31, could generate greater Ct oscillations in the determination of the test. This also agrees with the higher detection velocity for that combination.

In general, the amplification sensitivity decreased with increasing amounts of total DNA in the reaction. Thus, a reaction at concentrations above 250 ng of DNA decreased the probability of signal amplification for all the bacterial dilutions tested, in the range of 41.69% to 87.50%. Furthermore, in all cases of amplification at 500 and 700 ng of hDNA with combined DNA from *Brucella* and MTC, only amplicons belonging to the MTC were detectable. The amplification sensitivity was 100% with quantities of 100 and 250 ng of hDNA and coefficients of variation were always below 9%, variability consistent with that obtained from pure culture. These results are concordant with the study described by Morata et al. [20], where large amounts of human genomic DNA in PCR reactions can decrease the sensitivity of the assay due to their inhibitory effect.

The Tm and Ct values showed no significant differences between the different conditions for the amount of 150 ng of hDNA. For this reason, in the clinical phase we chose an amount of DNA per reaction not higher than 250 ng, amounts close to 150 ng of total DNA being preferred.

In general, the preliminary clinical results were very good. The three M RT-PCR assays selected in which the melting temperatures are clearly differentiated correctly identified all the samples and were negative for all the patients in the control group.

In conclusion, the presence of multicopy elements in multiplex real-time PCR reactions can affect the sensitivities and detection limits of the technique, which must be considered, together with the GC content, for the design of multiplex based-SYBR Green I assays. M RT-PCR assays based on the targets senX3-regX3+bcsp31 and senX3-regX3+IS711 using SG are highly reproducible and more sensitive than with other gene combinations. This may therefore be a promising and practical approach for the rapid differential diagnosis of extrapulmonary tuberculosis and complicated brucellosis. Further studies with a larger number of patients are needed to confirm this hypothesis.
